# Polymyxin B-immobilized fiber column hemoperfusion has the ability of endotoxin removal during 24 hours

**DOI:** 10.1186/cc10988

**Published:** 2012-03-20

**Authors:** C Mitaka, Y Ueda, Y Miyawaki, M Yamauchi, T Toyofuku, G Haraguchi, T Kudo

**Affiliations:** 1 Tokyo Medical and Dental University, Tokyo, Japan

## Introduction

Endotoxin plays an important role in the pathogenesis of septic shock. Endotoxin adsorption therapy by Polymyxin B-immobilized fiber column (PMX) hemoperfusion has been used for the treatment of septic shock patients in Japan. According to the company's recommendation, the standard duration of PMX treatment for patients with septic shock is 2 hours. However, we have shown that greater than 2 hours duration of PMX treatment significantly improved hemodynamics and significantly decreased administration of norepinephrine than 2 hours duration of PMX treatment. Our hypothesis was that PMX treatment had the ability of endotoxin removal during 24 hours. Therefore, the purpose of this study was to evaluate the endotoxin adsorption ability of 24 hours duration of PMX treatment.

## Methods

In this study, we measured plasma endotoxin concentrations of blood drawn from the radial artery and the outlet circuit of the PMX column after 24 hours duration of PMX treatment in septic shock patients. The assay for endotoxin was performed with separated plasma from heparinized whole blood samples centrifuged at 3,000 rpm for 40 seconds. The high-sensitivity assay was performed by kinetic turbidimetric Limulus assay using a MT-358 Toxinometer (Wako Pure Chemical Industries, Ltd, Japan). This Limulus assay test is specific to endotoxin and has no cross-reaction to β-glucan. The endotoxin removal rate was defined by the equation: ((radial artery endotoxin concentration - outlet circuit of PMX column endotoxin concentration)/radial artery endotoxin concentration) × 100%. The endotoxin removal rate represents endotoxin adsorption ability. Five patients with septic shock were studied.

## Results

The APACHE II scores of these patients were 26.2 ± 5.9 (mean ± SD, range 18 to 34) at admission to the ICU. Three patients survived and two patients died. Before the start of PMX treatment, heart rates were 119 ± 19 bpm, mean arterial pressures were 60 ± 19 mmHg, and plasma endotoxin concentrations of radial arterial blood were 91.4 ± 7.4 pg/ml (mean ± SD). After 24 hours duration of PMX treatment, plasma endotoxin concentrations decreased from 55.0 ± 58.9 pg/ml (radial arterial blood) to 19.4 ± 29.5 pg/ml (outlet circuit of PMX column). The endotoxin removal rate was 62.8 ± 22.1%, suggesting that endotoxin adsorption ability is still retained during 24 hours PMX treatment.

## Conclusion

These findings suggest that 24 hours duration of PMX treatment is effective to remove endotoxin. Further studies are needed to confirm this ability.
